# Fall Prevention in Community Settings: Results from Implementing Tai Chi: Moving for Better Balance in Three States

**DOI:** 10.3389/fpubh.2014.00258

**Published:** 2015-04-27

**Authors:** Marcia G. Ory, Matthew Lee Smith, Erin M. Parker, Luohua Jiang, Shuai Chen, Ashley D. Wilson, Judy A. Stevens, Heidi Ehrenreich, Robin Lee

**Affiliations:** ^1^Department of Health Promotion and Community Health Sciences, Texas A&M Health Science Center School of Public Health, College Station, TX, USA; ^2^Department of Health Promotion and Behavior, The University of Georgia College of Public Health, Athens, GA, USA; ^3^National Center for Injury Prevention and Control, Centers for Disease Control and Prevention, Atlanta, GA, USA; ^4^Department of Epidemiology and Biostatistics, Texas A&M Health Science Center School of Public Health, College Station, TX, USA; ^5^Department of Statistics, Texas A&M University, College Station, TX, USA

**Keywords:** Tai Chi: Moving for Better Balance, fall prevention, fall prevention program, community setting, older adults

## Abstract

Tai Chi: Moving for Better Balance (TCMBB) is an evidence-based fall prevention exercise program being disseminated in selected communities through state injury prevention programs. This study: (1) describes the personal characteristics of TCMBB participants; (2) quantifies participants’ functional and self-reported health status at enrollment; and (3) measures changes in participants’ functional and self-reported health status post-intervention. There were 421 participants enrolled in 36 TCMBB programs delivered in Colorado, New York, and Oregon. Of the 209 participants who completed both baseline enrollment and post-intervention surveys, the average age of participants was 75.3 (SD ± 8.2) years. Most participants were female (81.3%), non-Hispanic (96.1%), White (94.1%), and described themselves as in excellent or very good health (52.2%). Paired *t*-test and general estimating equation models assessed changes over the 3-month program period. Pre- and post-assessment self-reported surveys and objective functional data [Timed Up and Go (TUG) test] were collected. On average, TUG test scores decreased (*p* < 0.001) for all participants; however, the decrease was most noticeable among high-risk participants (mean decreased from 18.5 to 15.7 s). The adjusted odds ratio of reporting feeling confident that a participant could keep themselves from falling was five times greater after completing the program. TCMBB, which addresses gait and balance problems, can be an effective way to reduce falls among the older adult population. By helping older adults maintain their functional abilities, TCMBB can help community-dwelling older adults continue to live independently.

## Introduction

Tai Chi is a Chinese form of exercise that uses slow, flowing body movements. It had been practiced for centuries in Asia before being introduced to the United States in the early twentieth century (1). The physical and mental health benefits of Tai Chi are well documented (2–4), and in the 1990s, Tai Chi was rigorously tested by the National Institute on Aging as a fall prevention intervention (5, 6). A Cochrane review and meta-analysis concluded that Tai Chi reduced the risk of falling 28%, with greater effectiveness among those with lower initial fall risk (7). Today Tai Chi is widely recognized as an effective fall intervention (8–10).

The Tai Chi: Moving for Better Balance (TCMBB) program is an evidence-based fall prevention exercise program that was developed by researchers at the Oregon Research Institute with partial funding from the Centers for Disease Control and Prevention (CDC). The original 26-week intervention used 24 Tai Chi forms or sequences of controlled movements, and it was shown in a randomized controlled trial to be effective in reducing falls (11, 12). TCMBB consists of eight forms that progress from easy to difficult to improve older adults’ postural stability, balance, and coordination (13). Classes consist of 10–15 participants led by a trained instructor. One-hour classes are held twice a week for 12 weeks (24 total classes) (13). Feasibility testing has demonstrated that this program is well accepted by older adults and can be implemented with fidelity in community settings (14, 15).

In 2011, the CDC launched a 5-year project to implement TCMBB in selected communities in Oregon, Colorado, and New York. This was part of a larger project to reduce falls and fall-related injuries by engaging fall prevention coalitions, healthcare organizations, and other partners to integrate clinical and evidence-based community fall prevention programs in selected communities (16). TCMBB is intended for relatively healthy older adults with few functional limitations.

This study describes the results of implementing TCMBB during the first 2 years of the project. The purposes of the study were to: (1) describe the personal characteristics of TCMBB participants; (2) quantify participants’ functional and self-reported health status at enrollment; and (3) measure changes in participants’ functional and self-reported health status after completing the program.

## Materials and Methods

### TCMBB implementation

The three states offered TCMBB in a variety of settings including YMCAs, healthcare organizations, residential facilities, faith-based organizations, recreational facilities, and senior centers. State grantees hosted 30 TCMBB trainings from 2011 to 2013 at which Master Trainers from the Oregon Research Institute trained 400 instructors. In addition, the YMCA of the USA (Y-USA) engaged the Oregon Research Institute to train 10 YMCA faculty trainers to be TCMBB instructors.

The target audience for TCMBB is community-dwelling older adults aged 60 and older who can walk easily with or without assistive devices. In each state, participants were recruited by staff at member organizations, through family and friends, and through advertisements aimed at older adults. Methods of recruitment and referral varied across states and were based on existing partnerships. For example, Colorado and New York were most likely to recruit at YMCAs whereas Oregon recruited through senior centers and health care organizations since there were no YMCAs in their service delivery areas. As a program implemented through existing traditional community settings, there were limited exclusionary criteria and medical clearance was not required for participation. While no age restrictions were placed on enrollment, our analyses were restricted to people aged 60 years and older to reflect the study target population.

### Data collection

Data for this project were collected from multiple sources. Attendance was obtained from attendance logs collected at each class. A 20-question self-administered survey was used to collect pre- and post-TCMBB program data. The first was administered at the initial TCMBB class (enrollment or baseline survey) and the second at the final class (course completion or post-intervention survey). The surveys took approximately 15 minutes to complete and assistance was provided to participants who needed help filling out the forms. Questions included socio-demographic characteristics (e.g., age, sex, race, and ethnicity), whether the participant had been referred to the program by a healthcare provider, self-reported health status (excellent, very good, good, fair, or poor), satisfaction with their current activity levels (very, mostly, somewhat, or not at all satisfied), and confidence in their ability to keep themselves from falling (four-point scale ranging from strongly agree to strongly disagree). Self-reported functional ability was assessed by the reported level of difficulty in performing various activities (e.g., climbing one flight of stairs) on a four-point scale ranging from no difficulty (scored 1) to unable to do (scored 4) (17). Class completion was defined as attending at least 70% of the classes (i.e., 17 out of 24 classes).

The Timed Up and Go (TUG) test was used to measure functional status at enrollment and completion. This test has been widely used to assess functional mobility and predict fall risk and has been validated among community-dwelling older adults (18–20). The test measures the time in seconds required for participants to “rise from a standard arm chair, walk at [their] typical or normal pace to a line on the floor 3 meters away, turn, return, and sit down again” (21). Participants who completed the TUG in <12 seconds were classified as low risk and those who took 12 or more seconds were classified as high risk (22).

### Statistical analyses

Baseline characteristics (demographic characteristics, class attendance, and TUG results) were compared for those who completed both the baseline and post-intervention questionnaires to those who completed only the baseline questionnaire using chi-square tests to identify potential biases from loss to follow-up. Changes in TUG test times between baseline and post-intervention were compared using two-tailed paired *t*-tests; results were examined for all participants combined and stratified by baseline risk level. General estimating equations (GEE) models using a logit link function were used to compare differences in self-reported functional and health status at baseline and post-intervention; models were run using SAS version 9.3 GENMOD procedure (SAS Institute Inc., Cary, NC, USA) and adjusted for gender, age, race, and state. GEE models are longitudinal data models that use all available data in model estimation (i.e., do not require paired data) and can account for the correlation among repeated measures from the same participant (23).

The Texas A&M University Institutional Review Board granted approval to analyze data on program participants and outcomes collected using survey instruments and functional assessments.

## Results

### Participant characteristics and course attendance

Between September 1, 2011 and December 31, 2013, the three states offered 36 TCMBB programs and enrolled 537 people aged 60 and older. Of these enrollees, baseline data were collected from 421 (78.4%); 20.2% of participants were in Oregon, 39.9% in Colorado, and 39.9% in New York (Table 1). Of the 421 participants who provided a baseline questionnaire, 209 also completed a post-intervention questionnaire (Table 1).

**Table 1:** **Characteristics of Tai Chi: Moving for Better Balance (TCMBB) participants**.

	All enrolled participants^a^	Participants who completed both the baseline enrollment and post-intervention surveys	Participants who completed only the baseline enrollment survey	*X*^2^	*P*-value
	*N* = 421	*N* = 209	*N* = 212	
	*n* (%)	*n* (%)	*n* (%)	
Location				3.24	0.197
Oregon	85 (20.2)	35 (16.8)	50 (23.6)		
Colorado	168 (39.9)	85 (40.7)	83 (39.2)		
New York	168 (39.9)	89 (42.6)	79 (37.3)		
Age group				8.96	0.011
60–69	115 (27.3)	61 (29.2)	54 (25.5)		
70–79	177 (42.0)	98 (46.9)	79 (37.3)		
80+	129 (30.6)	50 (23.9)	79 (37.3)		
Gender				0.22	0.639
Female	335 (80.3)	169 (81.3)	166 (79.4)		
Male	82 (19.7)	39 (18.8)	43 (20.6)		
Missing	4	1	3		
Race				0.11	0.742
White	388 (93.7)	192 (94.1)	196 (93.3)		
Non-White	26 (6.3)	12 (5.9)	14 (6.7)		
Missing	7	5	2		
Ethnicity (Hispanic/Latino)				0.06	0.807
Yes	15 (3.7)	8 (3.9)	7 (3.4)		
No	395 (96.3)	198 (96.1)	197 (96.6)		
Missing	11	3	8		
Self-reported health status				3.86	0.145
Excellent/very good	211 (50.7)	108 (52.2)	103 (49.3)		
Good	165 (39.7)	85 (41.1)	80 (38.3)		
Fair/poor	40 (9.6)	14 (6.8)	26 (12.4)		
Missing	5	2	3		
Referred by healthcare provider					
Yes	35 (8.5)	18 (8.7)	17 (8.3)	0.03	0.872
No	378 (91.5)	189 (91.3)	189 (91.7)		
Missing	8	2	6		
Timed up and go (TUG) time at enrollment				5.65	0.017
Low risk (baseline TUG < 12 s)	279 (71.7)	154 (77.0)	125 (66.1)		
High risk (baseline TUG ≥ 12 s)	110 (28.3)	46 (23.0)	64 (33.9)		
Missing	32	9	23		
Participants who completed 70%+ classes	209 (49.6)	163 (78.0)	46 (21.7)	133.41	<0.001

*^a^Enrolled participants include all persons 60 years and older who filled out the baseline enrollment survey on the first day of the program (421/537 participants). Individual survey questions may have had missing data*.

The average age of participants was 75.3 (SD ± 8.2) years. Most participants were female, non-Hispanic, and White. About half of the participants attended at least 70% of classes (17 out of 24), with participants attending on average 13.6 (SD ± 8.0) of the 24 possible classes. Only 16 participants (8.5%) reported they were referred to TCMBB by a healthcare provider.

The 212 participants who “dropped out” or were lost to follow-up were not significantly different from those who completed the program in terms of gender, race, ethnicity, self-reported health status, or provider referral to class. However, dropouts were significantly older (average age 76.1 vs. 74.1) and more likely to have been classified as high risk based their TUG time at baseline.

### Participant functional performance

Of 421 participants with baseline data, 199 (47.3%) completed the TUG test at both baseline and post-intervention (Table 2). Of these, 45 (22.6%) were categorized as high risk. After completing TCMBB, the proportion of participants categorized as high risk decreased significantly to 14% (*n* = 28; data not shown). On average, TUG test scores decreased significantly for all participants but the change was most evident among high risk participants where the average TUG time decreased from 18.5 to 15.7 seconds.

**Table 2:** **Changes in Tai Chi: Moving for Better Balance (TCMBB) participants’ timed up and go (TUG) times from baseline enrollment to post-intervention^a^**.

Changes in TUG times (in seconds)	TUG at baseline		TUG at post-intervention		Change in TUG from baseline to post-intervention^b^		
	*N*	Mean (±SD)	*N*	Mean (±SD)	*N*	Mean (±SD)	*P*-value
TUG times for all participants	199	11.2 (±6.7)	199	9.9 (±6.0)	199	−1.3 (±2.7)	<0.001
Low risk (enrollment TUG time < 12 s)	154	9.1 (±1.4)	154	8.2 (±1.6)	154	−0.8 (±1.3)	<0.001
High risk (enrollment TUG time ≥ 12 s)	45	18.5 (±11.2)	45	15.7 (±10.5)	45	−2.7 (±4.9)	0.001

*SD, standard deviation*.

*^a^While 389 participants completed the TUG at enrollment, this table highlights the 199 participants who completed the TUG at both baseline and post-intervention*.

*^b^Paired *t*-tests with an alpha of 0.05 were used to compare changes in participants’ TUG times between baseline and post-intervention. A reduction in time indicates a positive functional improvement*.

### Self-reported outcome improvements

Table 3 compares self-reported outcome measures at baseline and post-intervention. Results are presented as percentages and as odds ratios adjusted for gender, age, race, and state. Significant improvements from baseline to post-intervention were observed for all outcomes except self-reported difficulty in walking across the room.

**Table 3:** **Changes in Tai Chi: Moving for Better Balance (TCMBB) participants’ self-reported health and functional outcomes from baseline to post-intervention**^a^.

Health outcome	Baseline (*N* = 209)^b^	Post-intervention (*N* = 209)^b^	Adjusted change from baseline to post-intervention^c^	
	*N* (%)	*N* (%)	Odds ratios from logistic models	*P*-value
Health status, satisfaction, and confidence				
Excellent or very good health status	108 (52.2)	123 (58.9)	1.35 (1.03, 1.77)	0.031
Very/mostly satisfied with physical activity levels	126 (60.9)	160 (76.9)	2.21 (1.60, 3.05)	<0.001
Feel confident not falling (strongly agree or agree)	149 (74.9)	196 (93.8)	6.16 (3.48, 10.89)	<0.001
Self-reported functional status				
No difficulty in walking across room	178 (86.4)	183 (88.4)	1.30 (0.83, 2.02)	0.249
No difficulty in walking one block	149 (77.2)	166 (83.0)	1.60 (1.19, 2.17)	0.002
No difficulty in stooping, crouching, and kneeling	71 (34.8)	85 (41.3)	1.32 (1.04, 1.68)	0.023
No difficulty in getting out of a straight back chair	141 (73.4)	163 (81.1)	1.67 (1.14, 2.44)	0.008
No difficulty in climbing one flight of stairs	133 (64.6)	144 (72.0)	1.42 (1.03, 1.68)	0.034

*SD, standard deviation*.

*^a^Data are reported for *n* = 209 participants who completed both the baseline and post-intervention surveys*.

*^b^The sample size is slightly smaller than 209 for some health outcomes due to missing data. The amount of missing data ranges from 0 to 8% for different outcomes*.

*^c^Adjusted odds ratios from GEE logistic regression modeling the probability of response = 1 at an alpha of 0.05. All models account for repeated measures from the same participant and are adjusted for gender, age, race, and state. An odds ratio >1 represents a positive improvement in self-reported health*.

The GEE model results showed that the adjusted odds ratio (aOR) of reporting excellent or very good health status increased by 35% (aOR = 1.35, 95% CI 1.03–1.77). The odds of being very or mostly satisfied with physical activity levels also increased significantly (aOR = 2.21, 95% CI 1.60–3.05). The odds of feeling confident that a participant could keep themselves from falling was five times greater after completing TCMBB (aOR = 6.16 95% CI 3.48–10.89).

Among the five items assessing functional status, the aORs for participants who reported “no difficulty” significantly increased for walking one block (aOR = 1.60, 95% CI 1.19–2.17); stooping, crouching, kneeling (aOR = 1.32, 95% CI 1.04–1.68); getting out of a straight back chair (aOR = 1.67, 95% CI 1.14–2.44); and climbing one flight of stairs (aOR = 1.42, 95% CI 1.03–1.68). About 86% of participants reported no difficulty walking across the room at baseline, and this proportion did not increase significantly at post-intervention.

## Discussion

This study examined 2 years of evaluation data collected from older adults age 60+ who participated in TCMBB programs offered in selected communities across three states. Comparing data collected at enrollment and course completion, TCMBB was associated with significant improvements in self-reported health status, satisfaction with physical activity levels, fall-related confidence, ability to perform basic functional tasks (e.g., walking one block, climbing a flight of stairs), and in the TUG test. Similar positive results have been seen in earlier studies of Tai Chi (14, 24), and provide additional evidence that Tai Chi is a useful fall prevention program for older adults.

Recruitment of participants is a concern for most fall prevention programs. While the distribution of TCMBB participants’ race and ethnicity was similar to the populations from which they were recruited, the percentage of male participants was low. Retaining TCMBB participants was also challenging. Participants attended on average 57% of the 24 classes. While it was not possible in this study to monitor falls, those who did not attend regularly may not have received an adequate intervention dose for reducing their fall risk. The reasons for low attendance are unknown. However, anecdotal reports from the state health departments implementing TCMBB suggest that some older adults may have considered the Tai Chi program a “drop-in” activity instead of an ongoing program. Those who did not complete the course were somewhat older and took longer to complete the TUG at enrollment, which suggests that health issues may have contributed to their not finishing the program.

Barriers to the success of TCMBB, as for other community-based fall prevention programs, include maintaining regular attendance and encouraging participants to continue activities after the program ends. Although the participants in this study demonstrated positive outcomes, one 12-week program is unlikely to provide long-term benefits without booster classes. Tai Chi, like other strength and balance exercises, is most effective when it is practiced for 50 hours or more (11). Therefore, older adults would benefit from having an ongoing Tai Chi program in their community, if they attended regularly. Some participating sites are now offering an introductory 12-week TCMBB followed by an ongoing program.

Another challenge has been the limited availability of community Tai Chi classes. State health departments have been able to address this by developing public–private partnerships with organizations that have existing infrastructure to offer classes to older adults. For example, the Y-USA now endorses a modified version of TCMBB called Y-Moving for Better Balance (Y-MFBB) that is being offered in local YMCAs (25). State health departments are also beginning to implement and support other Tai Chi programs (e.g., Tai Chi for Arthritis) that have been shown to be effective for fall prevention (26).

Ideally all older adults would have access to a wide range of evidence-based fall prevention programs that could meet their varied needs. Thus, in the larger fall prevention project, TCMBB was offered along with Stepping On (27) and Otago (28), which are designed for older adults with some functional limitations who are at moderate and high fall risk, respectively. As the availability of Tai Chi and other fall prevention programs expands, it will be important to ensure that fidelity to the key elements of the original interventions is maintained so that the programs remain effective in preventing falls.

### Limitations

This study has number of limitations. First, sampling and dropout issues limit the generalizability of the results. Participants were self-selected from participating communities and may not be representative of the older adult population either in those communities or in the participating states. Program effectiveness was based on comparing assessments from participants who attended both the first and last class. These participants were slightly younger and had fewer functional limitations, as measured by better TUG times at baseline, compared to participants who were not available for the post-intervention assessment. However, because the results are for those who provided both baseline enrollment and post-intervention assessments, we can be confident we are comparing the same population before and after the intervention. We did not take into account differences in total attendance among people who provided baseline and post-intervention assessments, so the effectiveness of the full intervention may be underestimated.

Second, the program was delivered in a multitude of settings, and outcomes may have been influenced by variability in instructor and site. Although all instructors were certified trained instructors, we recommend more attention be given to treatment fidelity monitoring in future research and practice. The CDC Guide for Program Implementation (13) has examples of a class observation form for monitoring instructor adherence to core program elements.

Third, in order to limit the reporting burden on the program delivery personnel, we used a limited number of self-reported outcomes and one timed functional assessment (i.e., the TUG). Although there was training provided for conducting the TUG, including available step-by-step online videos, this training was limited. Therefore, results may not be comparable to standardized TUG tests administered by trained professionals. Finally, while TCMBB participants reported improved functional status and demonstrated better TUG scores, we do not know if this led to a reduction in falls, since falls were not monitored during or after the program.

## Conclusion

Tai Chi: Moving for Better Balance, which addresses gait and balance problems, can be an effective way to reduce falls among the older adult population. Various forms of Tai Chi have been shown to be most appropriate for younger and healthier older adults who are at relatively low risk of falling. By helping older adults maintain their functional abilities, TCMBB can help community-dwelling older adults continue to live independently.

In this study, TCMBB participants reported positive effects on their functional and health status. However, the high dropout rates among program participants highlight a major challenge to implementing effective community-based fall prevention programs. Community-based programs are a promising approach for older adult fall prevention, but there are ongoing challenges to ensuring that high quality programs are available for – and attended by – older adults who can benefit from such programs.

## Conflict of Interest Statement

The findings and conclusions in this report are those of the authors and do not necessarily represent the official position of the Centers for Disease Control and Prevention.

This paper is included in the Research Topic, “Evidence-Based Programming for Older Adults.” This Research Topic received partial funding from multiple government and private organizations/agencies; however, the views, findings, and conclusions in these articles are those of the authors and do not necessarily represent the official position of these organizations/agencies. All papers published in the Research Topic received peer review from members of the Frontiers in Public Health (Public Health Education and Promotion section) panel of Review Editors. Because this Research Topic represents work closely associated with a nationwide evidence-based movement in the US, many of the authors and/or Review Editors may have worked together previously in some fashion. Review Editors were purposively selected based on their expertise with evaluation and/or evidence-based programming for older adults. Review Editors were independent of named authors on any given article published in this volume.
